# Occupational 4-Methylsulfonyl-benzonitrile poisoning: a case report

**DOI:** 10.3389/fphar.2025.1616964

**Published:** 2025-11-20

**Authors:** Jianjian Liu, Zhaozhao Shan, Yuru Liu, Yingying Zheng, Xiangdong Jian, Baotian Kan

**Affiliations:** 1 Department of Poisoning and Occupational Diseases, Emergency Medicine, Qilu Hospital of Shandong University, Cheeloo College of Medicine, Shandong University, Jinan, Shandong, China; 2 Department of Occupational and Environmental Health, School of Public Health, Cheeloo College of Medicine, Shandong University, Jinan, Shandong, China; 3 Department of Nursing, Department of gerontology, Qilu Hospital, Cheeloo College of Medicine, Shandong University, Jinan, Shandong, China

**Keywords:** 4-methylsulfonyl-benzonitrile, organic compound, toxic encephalopathy, toxic peripheral neuropathy, occupational exposure

## Abstract

**Background:**

4-Methylsulfonyl-benzonitrile is an organic compound used in organic synthesis with applications in medicine, dye, and pesticide production. However, its toxicological characteristics in humans remain poorly understood, with no previously reported cases of poisoning.

**Case presentation:**

This report describes a 33-year-old male who developed severe toxic encephalopathy and peripheral neuropathy following occupational exposure during herbicide production. The patient worked without adequate personal protective equipment in a workshop where he was intermittently exposed to organic solvents, including 4-methylsulfonyl-benzonitrile, over several months. He initially presented with dizziness, slurred speech, and mental deterioration, which progressed to impaired consciousness and respiratory failure requiring mechanical ventilation. Blood analysis revealed an initial 4-methylsulfonyl-benzonitrile concentration of 734 ng/mL upon initial testing, decreasing to 76 ng/mL, and becoming undetectable within 10 days. Magnetic resonance imaging showed diffuse symmetric abnormalities in the corpus callosum, bilateral basal ganglia, corona radiata, and centrum semiovale. Electromyography confirmed sensorimotor peripheral neuropathy.

**Intervention and outcome:**

The patient received comprehensive treatment including mechanical ventilation, organ protection, neurotrophic therapy, anti-infective therapy, and nutritional support. After 28 days of hospitalization, he was discharged with significant improvement—conscious, oriented, and with normalized speech, although mild numbness in the limbs persisted.

**Conclusion:**

This case demonstrates that 4-methylsulfonyl-benzonitrile poisoning can induce both toxic encephalopathy and peripheral neuropathy. The findings highlight an urgent need to strengthen safety monitoring and safety training in chemical manufacturing enterprises.

## 1 Introduction

4-Methylsulfonyl-benzonitrile is a versatile organic compound widely used in pharmaceutical synthesis, agrochemical production, and industrial applications. Despite its extensive use, its toxicological profile, particularly in occupational exposure, remains poorly understood. To date, no cases of human poisoning from this compound have been reported. Based on its chemical properties and analogy to other organic solvents, the theoretically expected manifestations of poisoning could include injuries to lipid-rich tissues, notably the brain and nervous system, as well as organs like the liver and kidneys (Argo et al., 2010). This report describes the first known case of severe toxic encephalopathy and peripheral neuropathy induced by occupational exposure to 4-methylsulfonyl-benzonitrile in a 33-year-old male herbicide production worker. The absence of prior clinical reports on the toxicology of the compound highlights the urgent need to elucidate its toxic mechanisms. Additionally, this case highlights the importance of occupational safety protocols, routine toxicological monitoring, and health management for workers exposed to chemical hazards.

## 2 Case description

A 33-year-old male, previously in good health, was admitted to Qilu hospital on 11 November 2024, following a diagnosis of chemical poisoning due to 4-methylsulfonyl-benzonitrile exposure for 20 days. The patient worked in a subordinate workshop at a pharmaceutical technology company in Jiangsu Province from 2023 to 2024, where he was involved in the production of raw herbicide materials. The workshop, approximately 400 m^2^, was equipped with industrial ventilation fans. However, the patient mostly worked without personal protective equipment and was intermittently exposed to various organic solvents, including benzonitrile. On 22 October 2024, he was admitted to a local hospital due to dizziness and speech disorder for 2 months, worsening for 5 days, along with mental abnormalities for 2 days. Cranial computed tomography (CT) and lumbar puncture revealed no obvious abnormalities. The local hospital provided symptomatic treatments, such as neurotrophic treatment, circulatory support, fluid infusion, antimicrobial therapy, organ protection, nutritional support, hemoperfusion, continuous renal replacement therapy, tracheal intubation, and mechanical ventilation. Toxicological analysis of the blood samples revealed 4-methylsulfonyl-benzonitrile levels of 734 ng/mL (24 October 2024), 76 ng/mL (27 October 2024), and undetectable levels by 1 November 2024. Despite treatment, the patient developed pneumonia caused by multidrug-resistant *Pseudomonas aeruginosa* and respiratory failure, making ventilator weaning difficult. For further diagnosis and treatment, he was transferred to Qilu hospital on 11 November 2024.

On arrival, the patient was in a comatose state and required mechanical ventilation. His vital signs were as follows: temperature (T), 37.7 °C; heart rate (HR), 118 beats/min; respiration rate (R), 15 beats/min; blood pressure, 158/86 mmHg. He exhibited profuse facial sweating but no jaundice or enlarged superficial lymph nodes. His pupils were equal in size, round (approximately 3 mm in diameter), and reactive to light. A subconjunctival hemorrhage was observed. His lips were not cyanotic, and the pharynx was non-congested. The neck was soft with no resistance. Bilateral thoracic motility was symmetrical, breath sounds were coarse, and wet rales were audible. The patient’s HR was 118 beats/min with a regular rhythm. No pathological murmurs were observed in valvular regions. The abdomen was flat with no intestinal pattern or peristaltic waves. Due to his condition, the patient was unable to cooperate during tenderness or limb muscle strength examinations. However, no deformities were observed in the spine or the extremities, and muscle tone was normal. Physiological reflexes were present, but no pathological reflexes were observed. Abnormal test results included blood routine: white blood cells 12.89 × 10^9^/L, neutrophil ratio 92.50%, neutrophil count 11.93 × 10^9^/L, plateletcrit 0.395%; procalcitonin 0.44 ng/mL; interleukin 6 (IL-6) 90.90 pg/mL; IL-1B 9.27 pg/mL, TNA-α 10.90 pg/mL; γ-Glutamyl transferase 95 IU/L, total bilirubin 29 μmol/L, unconjugated bilirubin 25 μmol/L; amylase 147 IU/L; and lipase 335.00 IU/L (Table 1). Diagnosis at admission included organic compound poisoning, pneumonia, and respiratory failure.

**TABLE 1:** The key laboratory parameters of the patient.

Items	Day1	Day3	Day7	Day14	Day21	Day28
WBC (3.5–9.5 × 10^9^/l)	**12.89**	**14.66**	**16.16**	**11.78**	**13.78**	6.69
NEU% (40.0%–75.0%)	**92.50**	**79.40**	**80.40**	70.20	**80.50**	57.40
HGB (115.0–150.0 g/L)	156	140	145	140	146	127
PLT (125–350 × 10^9^/l)	357	343	239	283	217	191
ALT (7.0–40.0 U/L)	30	18	12	34	20	30
AST (13.0–35.0 U/L)	43	28	14	21	18	16
γ-GT (10–60 U/L)	**95**	**69**	59	**103**	**85**	52
TBIL (5.0–21.0 μmol/L)	**29**	18.2	18.5	9.7	20.7	8.7
AMY (<105 U/L)	**147**	**141**	**277**	**279**	**222**	**163**
LIP (12–60 U/L)	**335**	**192**	**479**	**249**	**479**	**257**
Cr (53,0–97.0 μmol/L)	63	73	92	58	48	57
CK (38–174 U/L)	168	**476**	105	42	54	20
PT (8.8–13.8 s)	13.4	**18.4**	**18.1**	**14.7**	12.1	11.2
APTT (26.0–42.0 s)	**43.4**	36.6	35.5	31.1	31.5	29.8
PCT (<0.05 ng/mL)	**0.44**	—	—	—	—	—
IL-6 (0–7 pg/mL)	**90.90**	—	—	—	—	—

Day 1: Admission Day; WBC, white blood cell; NEU, neutrophils; HGB, hemoglobin; PLT, platelet; ALT, alanine aminotransferase; AST, aspartate aminotransferase; γ-GT, γ-Glutamyl Transferase; TBIL, total bilirubin; AMY, amylase; LIP, lipase; Cr, Serum Creatinine; CK, creatine kinase; PT, prothrombin time; APTT, Activated Partial Thromboplastin Time; PCT, procalcitonin; IL-6, Interleukin-6; The bold indicates laboratory parameters outside the normal range.

**FIGURE 1:**
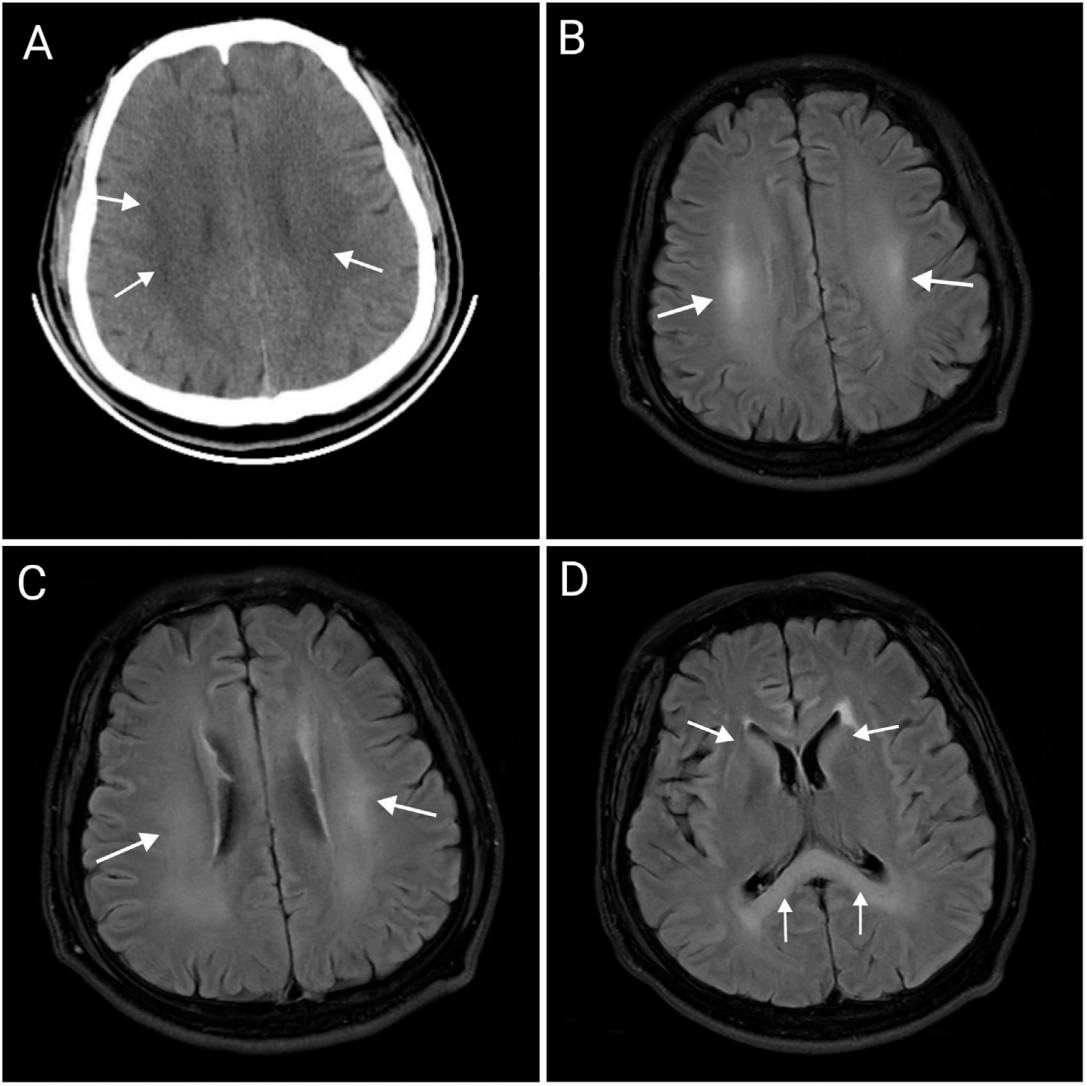
Abnormal imaging changes of the corpus callosum, bilateral basal ganglia, radiative crown, and hemi oval center **(A–D)**. Craniocerebral CT showing multiple patchy, low-density lesions with blurred margins in the brain **(A)**. MRI of the head showing hyperintensity on T2-FLAIR in the centrum semiovale **(B)**, corona radiata **(C)**, corpus callosum, and bilateral basal ganglia **(D)**, which suggests the possibility of toxic encephalopathy. CT, computed tomography; MRI, Magnetic resonance imaging.

**FIGURE 2:**
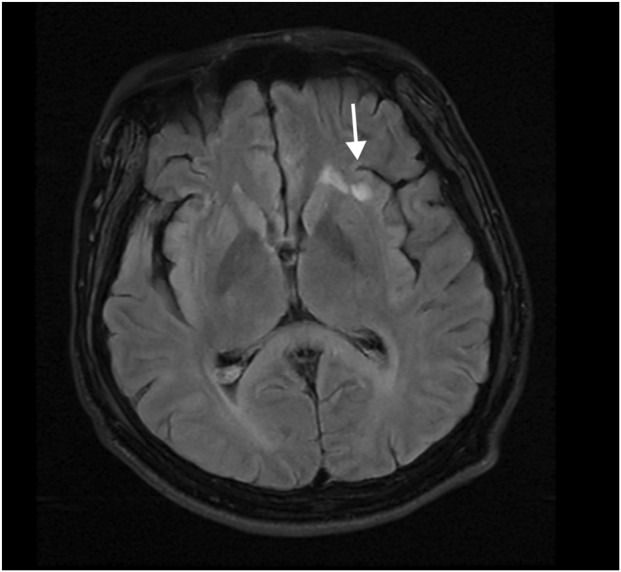
Abnormal signal in the left insular lobe of brain MRI. Patchy hyperintensity on T2-FLAIR in the left insular lobe, which requires observation during follow-up.

**FIGURE 3:**
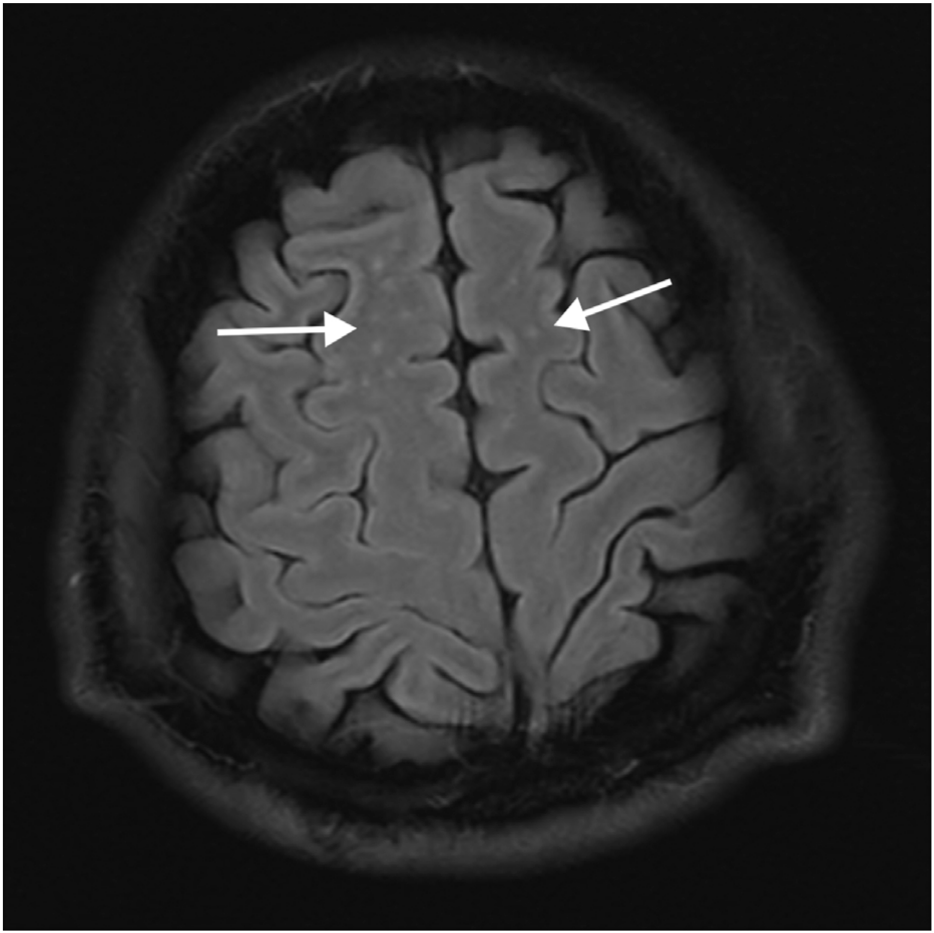
Abnormal signal in the bilateral frontal lobes of brain MRI. Multiple dot-shaped hyperintensity on T2-FLAIR in bilateral frontal lobe, which may represent an ischemic degenerative lesion.

**FIGURE 4:**
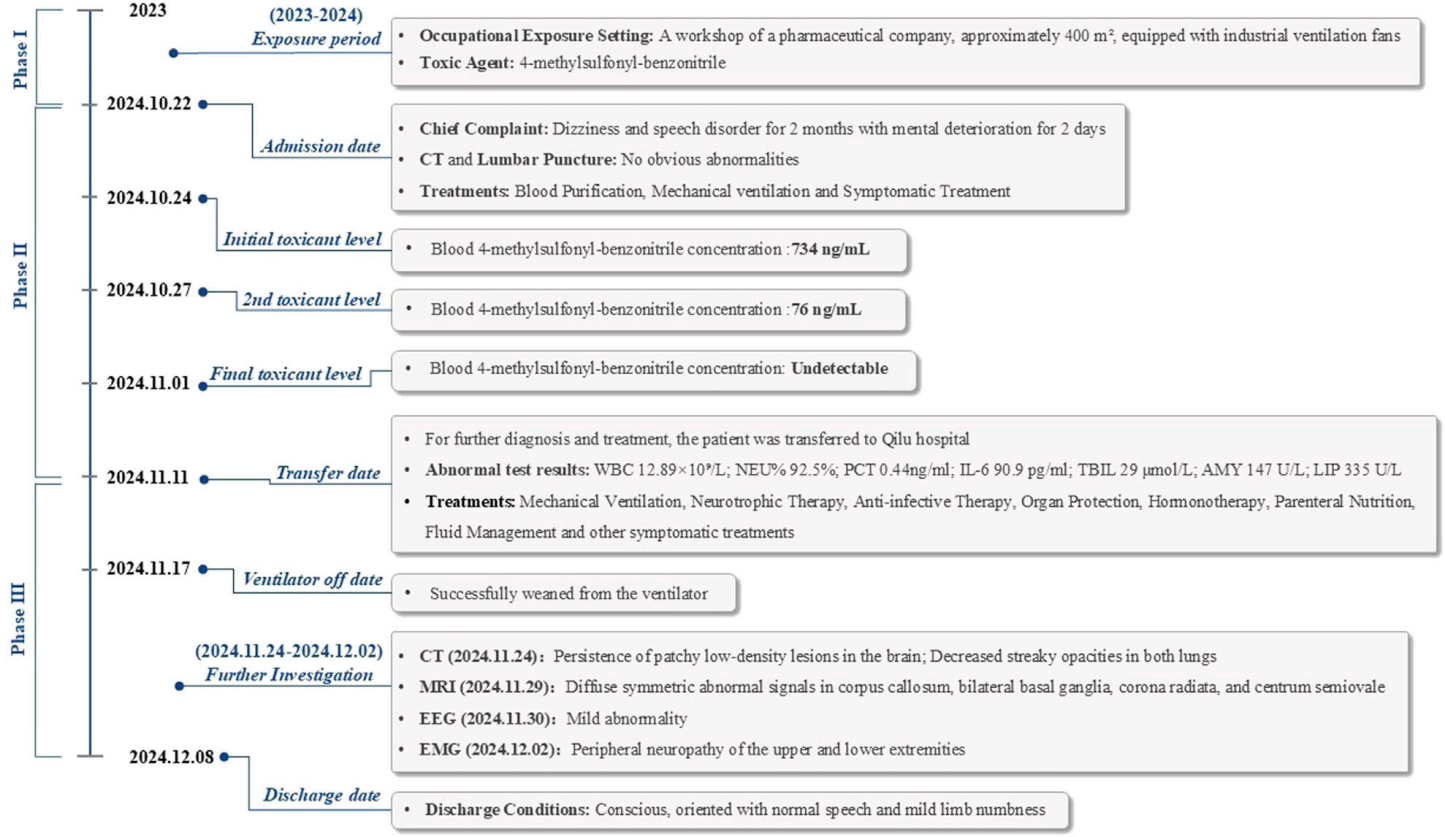
Timeline of the patient’s journey from exposure to treatment outcome.

After admission, the patient received ventilator-assisted ventilation, neurotrophic therapy (Mecobalamin Tablets, Vitamin B1 Injection), anti-infective therapy (Cefoperazone Sodium and Sulbactam Sodium for Injection), organ protection (Polyene Phosphatidylcholine Injection, Magnesium Isoglycyrrhizinate Injection, Furosemide Injection), Hormonotherapy (methylprednisolone sodium succinate for Injection), parenteral nutrition, fluid management and other symptomatic treatments. On the second day of admission, craniocerebral, thoracic, and abdominal CT revealed multiple patchy low-density lesions with blurred margins in the brain (Figure 1). The lung parenchyma exhibited increased and disordered pulmonary markings in both lungs, accompanied by high-density stripy shadows and ground-glass opacities. Bilateral pleural thickening was observed. On the fifth day after admission, the patient experienced intermittent convulsions, which were controlled with clonazepam. By the seventh day, the patient returned to spontaneous breathing and was successfully weaned off the ventilator. On the 14th day, the patient’s mental status improved, and he was able to follow verbal commands and nod in response. Repeat craniocerebral, thoracic, and abdominal CT scans demonstrated multiple persistent patchy low-density lesions in the brain, similar to those observed on 12 November 2024. The high-density, stripy shadows of the bilateral lungs remained but had decreased in size compared to the CT on 12 November 2024. Bilateral pleural thickening persisted. On the 19th day of admission, the patient’s mental status further improved, and he could follow instructions, although his speech remained dysarthria. Magnetic resonance imaging (MRI) of the head and neck revealed diffuse symmetric long T1 and T2 signals in the corpus callosum, bilateral basal ganglia, corona radiata, and centrum semiovale, with hyperintensity on T2-fluid-attenuated inversion recovery (FLAIR) and isointensity on diffusion-weighted imaging (DWI) (Figure 1). Patchy long T1 and T2 signal abnormalities were identified in the left basal ganglia and left insular lobe, showing hyperintensity on T2-FLAIR and isointensity on DWI (Figure 2). Multiple punctate long T1 and T2 signal shadows were found in the bilateral frontal lobes, showing hyperintensity on T2-FLAIR and isointensity on DWI (Figure 3). On the 20th day of admission, the electroencephalogram result showed a mild abnormality, with the background alpha rhythm reduced to 7–9 Hz, poor regulation, and amplitude modulation. Slightly more scattered slow activities were observed, mainly in the anterior head. On the 22nd day of admission, the electromyogram result indicated peripheral neuropathy of the upper and lower extremities (involving sensory and motor functions; sensory involvement was prominent, and axonal damage was likely). At the time of discharge on the 28th day of admission, the patient’s condition had significantly improved. He was conscious and oriented, with normal speech and appropriate responses to questions, although mild numbness in the limbs persisted. While this marked improvement is encouraging, the long-term neurological outcome following severe toxic encephalopathy and peripheral neuropathy remains guarded and requires continued monitoring. The patient was formally advised to seek specialized rehabilitation therapy to maximize functional recovery and was scheduled for regular outpatient follow-up to monitor for any potential long-term sequelae. The patient’s journey from exposure to treatment outcome is summarized in a timeline (Figure 4).

## 3 Discussion

4-Methylsulfonyl-benzonitrile is a key intermediate in organic synthesis, primarily used in pharmaceuticals, organic synthesis, dye production, pesticide production, and flavor production (PubChem, 2025). To date, there have been no case reports of 4-methylsulfonyl-benzonitrile poisoning. However, based on the chemical properties and clinical manifestations in patients, it can be preliminarily concluded that 4-methylsulfonyl-benzonitrile can cause toxic effects on the brain and peripheral nerves, leading to toxic encephalopathy and peripheral neuropathy. Toxic encephalopathy is an organic lesion of the central nervous system caused by toxins. Due to its lipophilic nature, 4-methylsulfonyl-benzonitrile can cross the blood–brain barrier and accumulate in the brain tissue, particularly in areas rich in lipids with a high metabolic rate (Dobbs, 2011). The clinical manifestations of toxic encephalopathy are nonspecific and mainly related to the involved brain regions (Kim and Kim, 2012). Mild poisoning may present with dizziness, headache, nausea, vomiting, and mild consciousness disorders, whereas moderate to severe poisoning may present with somnolence, delirium, coma, seizures, and brain herniation (GBZ 76–2024, 2024). In patients with toxic encephalopathy, MRI may show bilateral symmetrical and diffuse signal changes with white and gray matter involvement (De Oliveira et al., 2019). In the current case, the MRI findings showed significant involvement of the corpus callosum, bilateral basal ganglia, corona radiata, and centrum semiovale. Toxic peripheral neuropathy occurs when toxins damage peripheral nerves, affecting sensory, motor, and autonomic nerve fibers (Karam and Dyck, 2015). Mild poisoning may present with symmetrical stocking-like sensory reduction or hyperesthesia in the distal extremities and weakened physiological reflexes, while moderate to severe poisoning may present with muscle weakness in the extremities, deep sensory disturbances, and respiratory muscle paralysis (GBZ 76–2024, 2024). Electromyography, which records muscle action potentials, is useful for diagnosing peripheral neuropathy. The electromyography results of our patient indicated peripheral neuropathy in both the upper and lower extremities (involving both sensory and motor nerves, with the sensory nerves being more affected and axonal damage likely), suggesting that 4-methylsulfonyl-benzonitrile causes physical lesions in the peripheral nerves.

Placing our findings in the context of other solvent poisonings helps delineate the neurotoxic characteristics of 4-methylsulfonyl-benzonitrile. The toxic encephalopathy observed in our patient, evidenced by cognitive disorder and impaired consciousness, shares features with other organic solvent poisonings. For instance, the widespread white matter involvement on MRI is a hallmark of chronic toluene leukoencephalopathy (Zeng et al., 2014). However, the objective evidence of peripheral neuropathy in our case is not typical of toluene, which rarely causes this condition (Hormes et al., 1986). Compared to the toxic encephalopathy caused by 1,2-dichloroethane (DCE), which is characterized by extensive but often reversible brain edema (Liu et al., 2010), our patient exhibited persistent MRI changes, suggesting a component of structural damage that is currently irreversible. In conclusion, while the clinical manifestations of organic compound poisoning can share common features, they often vary depending on the specific compound involved, its physicochemical properties, and its mechanism of toxicity. When diagnosing organic compound poisoning, it is essential to consider clinical manifestations, auxiliary examinations, occupational exposure history, and toxicant levels in blood and urine samples to reduce the probability of misdiagnosis. Due to the lack of specific antidotes, there is currently no specific treatment for 4-methylsulfonyl-benzonitrile poisoning, and treatment mainly focuses on symptomatic and supportive care.

From the perspective of public health and occupational health, this case highlights the urgent need to strengthen systemic protections in work environments where toxic chemicals are produced. To effectively prevent the recurrence of such occupational poisoning incidents, we recommend that the relevant enterprises strengthen oversight of production environments. This should include conducting regular occupational safety assessments and performing concentration monitoring for the toxic substances to ensure the effective operation of protective systems. On the other hand, strengthen safety training for employees, particularly new hires, to foster a positive safety culture that encourages workers to proactively report any symptoms of discomfort. Furthermore, it is essential to establish detailed emergency response plans for occupational exposure incidents, ensuring that medical rescue can be promptly initiated.

This research, however, is subject to several limitations that should be acknowledged. First, the detailed clinical data from the patient’s initial presentation and early management at the local hospital were not available for review, which precludes a more granular analysis of the symptom onset. Second, given that this is the first reported case of human poisoning, there are no prior studies in the literature to establish a blood concentration-effect relationship. The blood concentrations measured in this patient (734 and 76 ng/mL) represent isolated data points from a single severe exposure. Therefore, these values cannot be extrapolated to define thresholds for mild or moderate poisoning. Future studies that accumulate data from additional cases will be essential to delineate the compound’s toxicological characteristics and correlate blood levels with clinical severity.

## Data Availability

The original contributions presented in the study are included in the article/supplementary material, further inquiries can be directed to the corresponding authors.
